# Screening Genotoxicity Chemistry with Microfluidic Electrochemiluminescent Arrays

**DOI:** 10.3390/s17051008

**Published:** 2017-05-03

**Authors:** Itti Bist, Kiran Bano, James F. Rusling

**Affiliations:** 1 Department of Chemistry, University of Connecticut, Storrs, CT 06269, USA; Itti.bist@Uconn.edu (I.B.); kiran.bano@uconn.edu (K.B.); 2 Department of Surgery and Neag Cancer Center, University of Connecticut Health Center, Farmington, CT 06032, USA; 3 Institute of Material Science, University of Connecticut, Storrs, CT 06269, USA; 4 School of Chemistry, National University of Ireland at Galway, Galway, Ireland

**Keywords:** electrochemiluminescence, genotoxicity, arrays, cytochrome P450, microfluidics

## Abstract

This review describes progress in the development of electrochemiluminescent (ECL) arrays aimed at sensing DNA damage to identify genotoxic chemistry related to reactive metabolites. *Genotoxicity* refers to chemical or photochemical processes that damage DNA with toxic consequences. Our arrays feature DNA/enzyme films that form reactive metabolites of test chemicals that can subsequently react with DNA, thus enabling prediction of genotoxic chemical reactions. These high-throughput ECL arrays incorporating representative cohorts of human metabolic enzymes provide a platform for determining chemical toxicity profiles of new drug and environmental chemical candidates. The arrays can be designed to identify enzymes and enzyme cascades that produce the reactive metabolites. We also describe ECL arrays that detect oxidative DNA damage caused by metabolite-mediated reactive oxygen species. These approaches provide valuable high-throughput tools to complement modern toxicity bioassays and provide a more complete toxicity prediction for drug and chemical product development.

## 1. Introduction

Toxic chemicals and their metabolites generated by cytochrome P450 (cyt P450) enzymes and bioconjugate enzymes can cause DNA damage. Various types of DNA damage can be caused by toxic compounds, including oxidation of DNA bases, nucleobase adduct formation, depurination of adducts, and strand breaks [1]. DNA oxidations can occur from ionizing radiation and reactive oxygen species (ROS) [2,3,4] with the major product 8-oxo-2-deoxyguanosine (8-oxodG) on DNA [5] being responsible for mutagenesis. DNA adducts formed by coupling of reactive metabolites and DNA bases are major biomarkers for cancer risk in humans, so a thorough understanding of reactive metabolites of chemicals generated by cyt P450s [6] and bioconjugation enzymes is essential for a complete toxicity pathway assessment of chemicals and drugs.

Electrochemical methods for DNA damage and toxicity detection were pioneered by Paleček [7] and Fojta [8]. To date, various electrochemical sensors for DNA damage, DNA interactions with drugs, environmental pollutants, and other potentially genotoxic species have been developed and reviewed by various authors [9,10,11,12,13,14]. However, our group is the only one to develop metabolite-generating microfluidic electrochemiluminescent (ECL) arrays for predicting metabolic genotoxicity chemistry of xenobiotics. This review focuses on our ECL sensor technology for genotoxicity screening.

Our approach as described herein features ECL arrays comprised of microwells containing dense DNA/enzyme films that produce metabolites in close proximity to DNA. ECL dyes that utilize damaged DNA as a co-reactant are included in the microwells. Extension of reactive metabolite studies to complementary LC-MS/MS analyses can be done utilizing DNA/enzyme films on magnetic beads to biocolloid enzyme reactors [15,16,17,18]. This review describes the sensor arrays, chemistry, and mechanisms of genotoxicity revealed using these ECL arrays.

## 2. Metabolic Pathway Involved in Genotoxicity

**Scheme 1:**
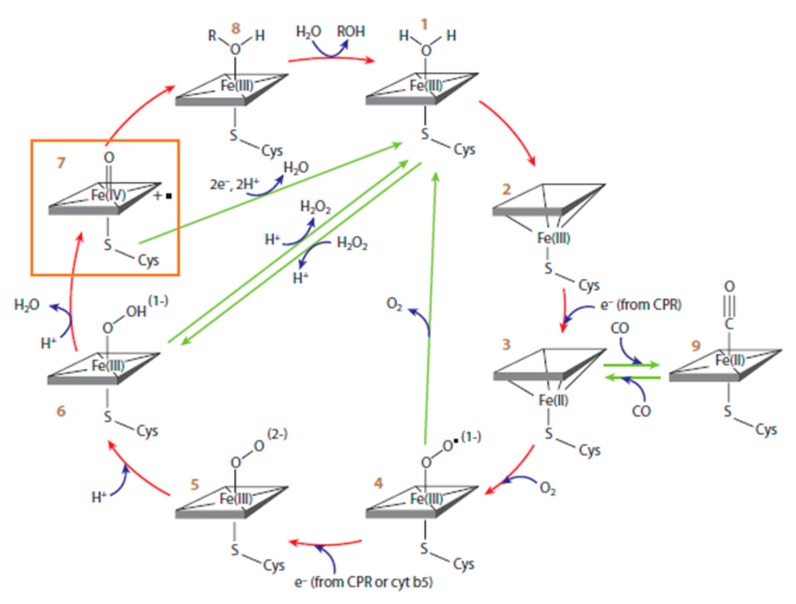
The proposed catalytic pathway for cyt P450-catalyzed metabolic reactions [17,25].

Cyt P450s are important metabolic enzymes that catalyze 75% of all known metabolic reactions involving drugs and other xenobiotics and can often generate reactive metabolites [19,20]. These reactive metabolites can cause DNA damage, and the parent chemicals are then described as *genotoxic*. Cyt P450-catalyzed metabolic pathways works through a complex catalytic cycle by involving the ferric iron–heme (P-Fe) prosthetic group. A two-electron reduction of the heme iron breaks the oxygen-Fe^III^ bond using electrons shuttled by cyt P450 reductase (CPR) from NADPH to cyt P450s. Reduction of cyt P450-Fe^III^ and protonation yields the cyt P450-Fe^III^-hydroperoxo complex **6** (Scheme 1). This complex then produces an active ferryloxy species (cyt P450-Fe^IV^=O, **7**) that transfers oxygen to bound substrate (RH) to oxidize it, often by an oxygen transfer reaction. The oxidized group can then serve as a site for attaching a solubilizing conjugate. Our genotoxicity assays can incorporate any human cyt P450s and/or metabolic bioconjugation enzymes to mimic these events in the human body [15]. Many substrates yield DNA-reactive metabolites, including styrene, polyaromatic hydrocarbons, nitrosamines, aromatic amines, tamoxifen, and other chemotherapeutic agents [21,22,23,24].

## 3. Development of ECL-Based Genotoxicity Sensors

**Scheme 2:**
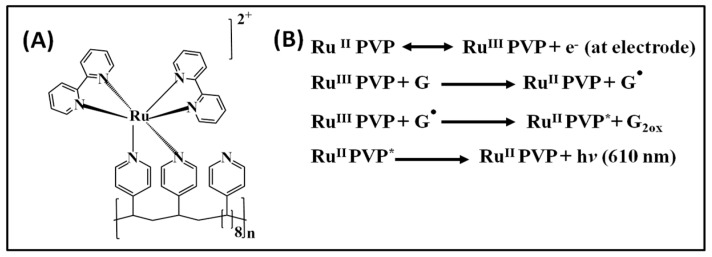
(**A**) Structure of ruthenium polyvinylpyridine (RuPVP); (**B**) proposed reaction mechanism for electrochemiluminescent (ECL) generation using RuPVP and guanines in DNA.

**Scheme 3:**
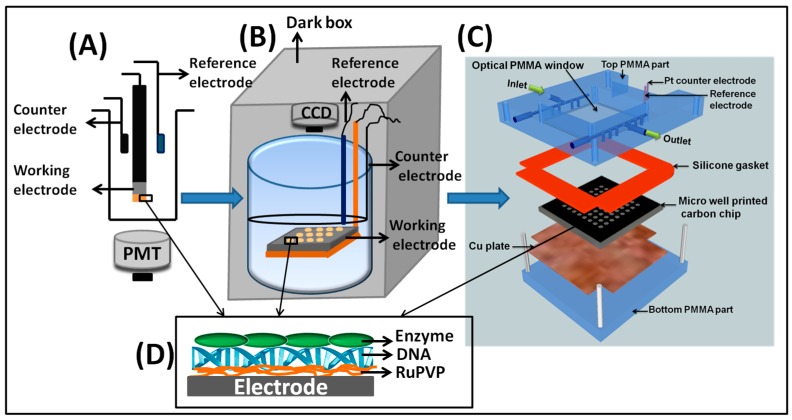
Evolution of ECL genotoxicity sensors, starting from a single sensor to a 64-microwell array. (**A**) A single-electrode sensor coated with RuPVP/enzyme/DNA films as shown in (**D**). The sensor was placed above an optical fiber connected to a photomultiplier tube (PMT) for ECL detection. (**B**) Array showing a pyrolytic graphite (PG) chip working electrode with the RuPVP/enzyme/DNA film spots shown with Ag/AgCl reference and Pt-ring wire counter electrode. After the enzyme reaction driven by NADPH, for ECL readout, the device is placed in a dark box and 1.2 V are applied while a charge-coupled device (CCD) camera measures ECL. (**C**) Recent ECL microwell PG chip in a microfluidic reaction chamber with symmetrically placed reference and counter electrodes on the underside of the top plate. Reactants flow into the chamber, and ECL is then captured by using a CCD camera in a dark box.

Our early toxicity sensors combined metabolic enzymes, DNA, and polyions in layer-by-layer (LBL) thin films on graphite electrodes through electrostatic adsorption of alternately charged layers of a redox ECL polymer, DNA, enzymes, and synthetic polyions [26]. Metabolites are produced in an enzyme reaction step, and DNA damage is subsequently monitored using ECL and LC-MS bioreactors [27,28]. The ECL dye polycation (bis-2,2′-bipyridyl) ruthenium polyvinylpyridine ([Ru(bpy)_2_(PVP)_10_]^2+^ or RuPVP) is oxidized by the electrode at ~1.2 V vs. Ag/AgCl to yield Ru^III^-PVP, which catalytically oxidizes guanines to guanine radicals (G^•^). G^•^ in intact DNA serves as an ECL co-reactant to eventually produce excited Ru^2+^*-PVP, which emits light at 610 nm (Scheme 2). Initially, single electrode ECL sensors coated with RuPVP/enzyme/DNA were developed as shown in Scheme 3A. These sensors in some cases measured ECL and voltammetry simultaneously [29,30].

We also demonstrated ECL from oxidized DNA in ultrathin films of cationic polymer [Os(bpy)_2_(PVP)_10_]^2+^ [PVP = poly(vinyl pyridine)] or OsPVP utilizing 8-oxodG as the co-reactant [31]. OsPVP combined with RuPVP were assembled into films with DNA or oligonucleotides to detect both DNA oxidation and nucleobase adducts from chemical damage. Electrochemically oxidized Os^II^ sites were generated from films containing 8-oxodG on DNA formed by chemical oxidation using a Fenton reagent that produced ECL specific for oxidized DNA, and Ru^II^ sites gave ECL from chemically damaged DNA, and possibly from cleaved DNA strands [28].

**Scheme 4:**
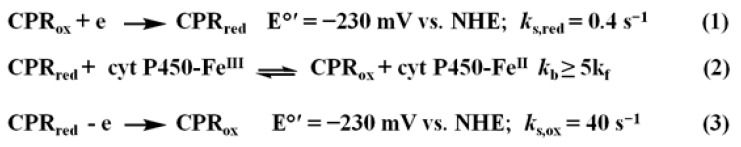
E_r_CE_o_ simulation model for cyclic voltammetry (CV) of cyt P450s + cyt P450 reductase (CPR).

More recently, we have incorporated microsomal CPR/cyt P450 films on electrodes to mimic the situation in vivo. Cyclic voltammetry (CV) of this system closely mimics the details of the natural cyt P450 catalytic pathway. As in Scheme 1, electrons flow from the electrode to CPR and then to the cyt P450 as in the in vivo pathway to facilitate efficient catalytic turnover of the cyt P450s [32]. CPR is reduced by the electrode but cyt P450s are not (Scheme 4, Equation (1)). CPR_red_ exists in redox equilibrium with cyt P450Fe^III^ to yield CPR_ox_ as a product (Scheme 4, Equation (2)), and electrochemical oxidation of CPR_red_ (Scheme 4, Equation (3)) competes with this equilibrium. We later found that a similar catalytic mechanism can be activated in microsomes containing multiple cyt P450s and CPRs [26,33,34]. Thus, the microsomal CPR-containing film is a model for the natural cyt P450 catalysis pathway [30] and can be used in toxicity sensors to electrochemically active cyt P450s.

Catalytic oxidation of 4-(Methylnitrosamino)-1-(3-pyridyl)-1-butanone (NNK) with the CPR/cyt P450 films further confirmed the electrode CPR cyt P450 pathway for activation of cyt P450s [32,33]. The formation rate of the product of this reaction in bulk electrolysis was slightly larger than that achieved in the same system using NADPH as electron donor. CV simulations (Scheme 4, Equation (1)) suggest an equilibrium complex between CPR and cyt P450 that facilitates electron transfer [35]. The theoretical data (*k*_b_ ≥ 5*k*_f_ and *K* = *k*_f_/*k*_b_ ≤ 0.2, where *k*_f_ and *k*_b_ are forward and backward second-order chemical rate constants and *K* is the equilibrium constant) lies in favor of CPR_red_, which is why CPR_ox_ is reduced electrochemically in cyt P450/CPR films that are preferential to cyt P450. The difference in formal potential (Δ*E*°) between CPR and cyt P450 is Δ*E*° = [*RT/nF*] ln(*K*), where *R* is the gas constant, *T* is the absolute temperature, and *F* is Faraday’s constant. Thus, *K* < 0.2 predicts that cyt P450 formal potential is ~40 mV negative of CPR_ox_ in the films, so that CPR_ox_ is more easily reduced. O_2_ present in catalytic oxidations reacts with P450-Fe^II^ and the equilibrium shifts to the right to drive substrate oxidation [32].

## 4. Microfluidic ECL Arrays for Genotoxicity Screening

ECL-based genotoxicity sensor arrays were first developed in our laboratory in 2007. These arrays were fabricated on a single conductive chip instead of multielectrode devices. In these ECL arrays, a 1-inch^2^ (6.45 cm^2^) pyrolytic graphite (PG) block was electrically connected to a copper block via silver epoxy and insulated using ethyl acrylate so only the upper conductive PG face was exposed. RuPVP/enzyme/DNA films were spotted on demarcated locations. Metabolite generation was driven by manual deposition and incubation of reactants on the arrays (Scheme 3B), using an NADPH regeneration system to drive cyt P450 catalysis.

These ECL arrays eventually led to our current high-throughput arrays integrated into a microfluidic reactor. A 64-microwell pattern was computer laser-jet printed and transferred onto a cleaned and polished PG chip (2.5 × 2.5 × 0.3 cm) by heat pressing the mask onto the PG chip at 290 °F (143 °C) for 90 s. The hydrophobic toner separating the spots prevents cross-contamination of DNA, enzyme and polymer solutions deposited in each well for LBL film formation. Individual RuPVP/enzyme/DNA spots were constructed in each microwell by sequential depositions of 1 μL drops of the appropriate solution. Then, the PG chip was fitted into a microfluidic reaction cell consisting of top and bottom poly(methylmethacrylate) (PMMA) plates with an optical glass window to facilitate ECL light acquisition. Pt counter and Ag/AgCl reference electrode wires were fixed on the bottom side of the top PMMA plate. A silicone rubber gasket sandwiched between the PMMA plates was used to form a sealed channel, and a copper plate was placed underneath the PG chip for electrical connection. The microfluidic device was connected to a syringe pump for a constant flow of reactants or buffers at a fixed concentration and flow rate to better control reaction conditions in the semi-automated system (Scheme 3C). Metabolites were generated in situ by flowing test compounds through the chip, and ECL was then generated by applying a potential of 1.25 V vs. Ag/AgCl for 180 s and captured by a CCD camera in a dark box.

**Scheme 5:**
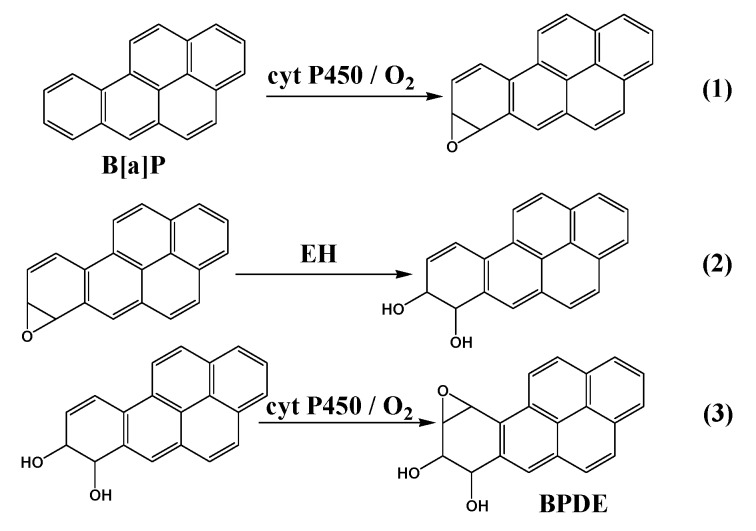
Metabolic activation of B[a]P by cyt P450 and epoxide hydrolase (EH).

Our first task for this microfluidic reactor focused on reactive metabolites of benzo[a]pyrene (B[a]P). Cyt P450 enzyme sources included rat liver microsomes (RLM) and human liver microsomes (HLM), supersomes (genetically engineered to contain only one cyt P450 + CPR) of cyt 1B1, 1A1, and 1A2 and human liver S9 fraction. B[a]P was initially oxidized by cyt P450s to B[a]P-7,8-epoxide (Scheme 5, Equation (1)), which was then hydrolyzed to B[a]P-7,8-dihydrodiol in presence of epoxide hydrolase (EH, Equation (2)). Further oxidation of dihydrodiol by cyt P450 resulted in the formation of reactive metabolite B[a]P-7,8-dihydrodiol-9,10-epoxide (BPDE, Equation (3)), which reacts with guanine and adenine bases in DNA to form adducts.

**Figure 1:**
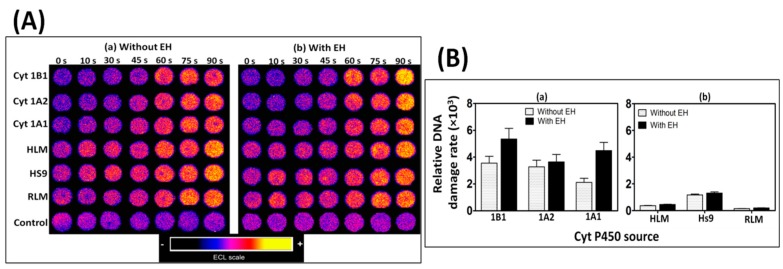
(**A**) Recolorized and reconstructed ECL array results for enzyme reactions with oxygenated 25 µM B[a]P + 1% DMSO in pH = 7.4 phosphate buffer with electronic activation of cyt P450s. Microwells containing RuPVP/enzyme/DNA film assemblies exposed to B[a]P for various reaction times. Enzyme sources are cyt P450 supersomes, human, and rat liver microsomes (HLM and RLM), epoxide hydrolase (EH), and human S9 fractions (HS9). (**B**) Influence of EH on the sensor array, relative DNA damage rate for 25 μM B[a]P at pH 7.4: (**a**) supersomes; (**b**) microsomes. Reproduced from [36] with permission, copyright Royal Society of Chemistry, 2013.

ECL is normalized for the amount of enzyme in each film (estimated by quartz crystal microbalance) to find the relative rate of DNA damage by B[a]P metabolites. The relative rate of DNA damage was higher when EH was included with cyt P450 in microwells when using HLM, RLM, or supersomes, suggesting a complete bioactivation of B[a]P to produce DNA-BPDE adducts. Cyt P450 1B1 was found to have the highest rate of DNA damage followed by cyt P450s 1A1 and 1A2 (Figure 1). The formation of DNA adducts in the presence of B[a]P was confirmed by liquid chromatography–mass spectrometry (LC-MS/MS) by determining dG-BPDE and dA-BPDE adducts using similar films of DNA/enzyme on magnetic bead reactors. The ECL arrays can also measure enzyme inhibition, in this case by measuring a decrease in the %ECL corresponding to a decrease in DNA damage, in the presence of furafylline and rhapontigenin, which are known inhibitors of cyt 1A2 and 1A1, respectively [36].

## 5. ECL Arrays for Assessing Organ-Specific Metabolic Toxicity

**Scheme 6:**
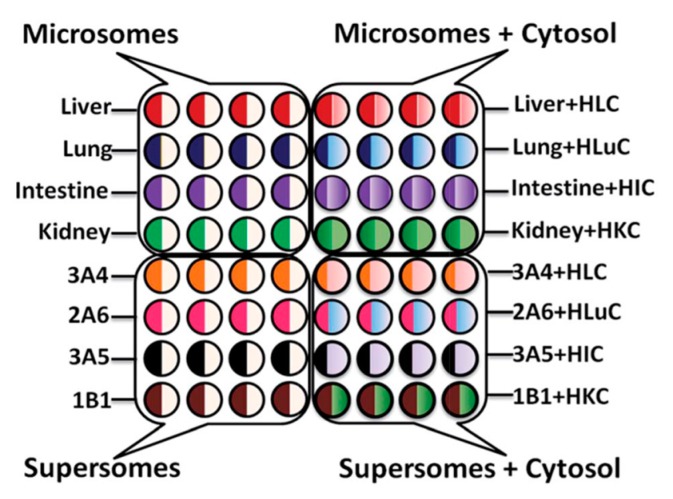
Experimental design of 64-microwell ECL chip with films of RuPVP/enzymes/DNA for metabolic genotoxicity assays of test compounds. Symbols: H = human; L = liver; C = cytosol; Lu = lung; I = intestine; K = kidney; numbers and letters such as 3A4 indicate supersomes containing the denoted single cyt P450 manifold. Reproduced from [37] with permission, copyright Royal Society of Chemistry, 2015.

Traditional toxicity assessment focuses on the liver, however, extra-hepatic tissues are also capable of metabolizing xenobiotics into reactive metabolites. The 64-microwell ECL array described above was further developed to evaluate possible genotoxic chemistry effect with different organ enzymes. Enzymes from human liver, lung, intestine, and kidney were compared in the array (Scheme 6).

**Figure 2:**
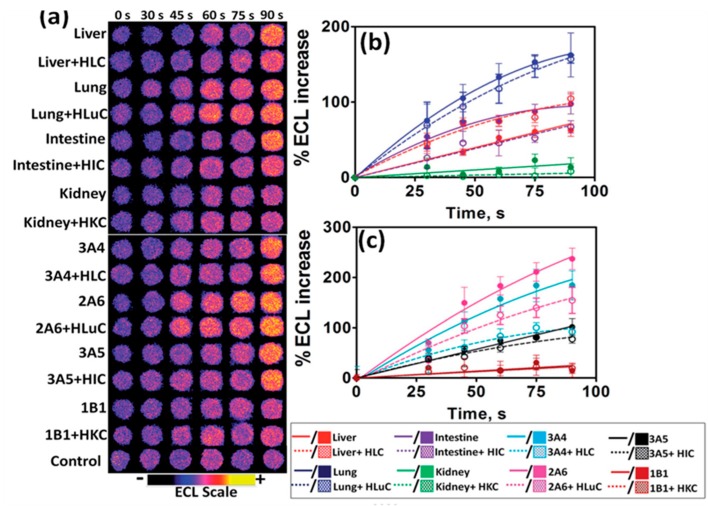
ECL results from genotoxicity study of oxygenated 150 μM NNK in pH 7.4 phosphate buffer and necessary cofactors exposed to microwells containing RuPVP/enzyme/DNA with electrochemical activation of cyt P450s at −0.65 V vs. Ag/AgCl (0.14 M KCl) for different reaction times. (**a**) Reconstructed, recolorized ECL array images. Control spots contain microsomes exposed to the same reaction conditions as above without exposure to NNK. Graphs show influence of enzyme reaction time on %ECL increase for reaction with 150 µM NNK at pH = 7.4, (**b**) with human organ tissue enzymes, and (**c**) with individual cyt P450 supersomes. Reproduced from [37] with permission, copyright Royal Society of Chemistry, 2015.

4-(Methylnitrosamino)-1-(3-pyridyl)-1-butanone (NNK), a known carcinogen, was used as a test compound to study the organ-specific metabolic-DNA reactions for enzymes from the different organs (Figure 2). A decrease in relative DNA damage rates in the presence of cytosolic enzymes and their co-factors were observed, which is consistent with the detoxification effect of cytosols via various bioconjugation pathways [37].

The two-tier approach based on ECL array and LC-MS/MS biocolloid reactors provided a more detailed profile of possible genotoxicity pathways associated with specific organs. Correlations between DNA damage rates obtained from the cell-free ECL arrays and cell-based Comet assays that measure DNA damage in cells were found [37].

## 6. ECL Sensor Arrays for DNA Oxidation

Oxidative stress or oxidative DNA damage is an imbalance between the production of ROS and the ability of the body to counteract their harmful effects [38]. ROS oxidizes guanosines (dG) in the DNA to form 8-oxodG, a biomarker for oxidative DNA damage [39]. 8-OxodG serves as a co-reactant for Osmium complex [Os(bpy)_2_(phen-benz-COOH)]^2+^ {bpy = 2,2′-bipyridine; phen-benz-COOH = (4(1,1phenanthrolin-6-yl)benzoic acid)}. ECL is generated due to selective catalytic oxidation of 8-oxodG by Os^III^ sites in the polymer, leading to the formation of a photoexcited Os^II^* species that emits ECL [31]. The 64-microwell array described in the above section was used to develop an ECL array for oxidative DNA damage.

**Scheme 7:**
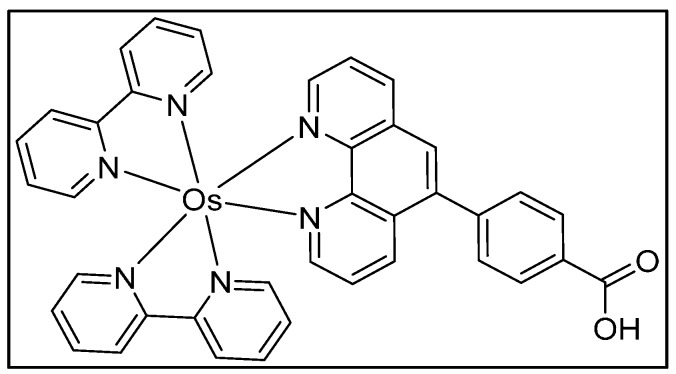
Structure of [Os(bpy)_2_(phen-benz-COOH)]^2+^.

**Figure 3:**
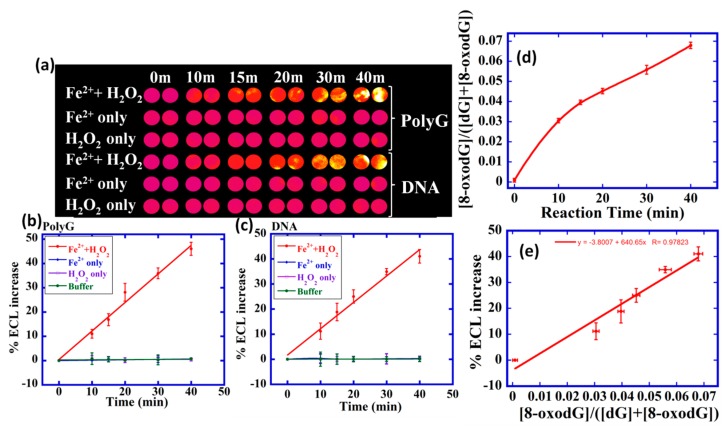
ECL array data for polyG and DNA oxidation. (**a**) Reconstructed ECL array data demonstrating ECL from spots of OsNG/polyG or DNA film treated with Fenton’s reagent in pH 7.2 phosphate buffer saline for various time intervals. Controls are polynucleotides treated only with buffer, with only FeSO_4_ or with only H_2_O_2_. Graphs show relative %ECL increase for (**b**) polyG and (**c**) DNA. (**d**) UHPLC-MS/MS for ECL array calibration showing measured ratio of [8-oxodG]/([dG]+[8-oxodG]) for DNA reacted with Fenton’s reagent for different time and then hydrolyzed. (**e**) Calibration plot for %ECL increase vs. relative amount of 8-oxodG. Reprinted with permission from [40], copyright (2016), American Chemical Society.

8-oxodG was detected in intact ds-DNA using composite films of [Os(bpy)_2_(phen-benz-COOH)]^2+^ (Scheme 7), Nafion polymer, and reduced graphene oxide (OsNG) [40]. A homogeneous solution of [Os(bpy)_2_(phen-benz-COOH)]^2+^-Nafion was drop-casted into the microwells of the PG chip array containing a layer of reduced graphene oxide, followed by the deposition of oxidized DNA solution on the OsNG-modified films. The PG chip is placed in the microfluidic device and washed, and ECL is then captured using a CCD camera while applying 0.9 V vs. Ag/AgCl. Fenton's reagent was used as a model compound for DNA and polydeoxyguanosine (polyG) oxidation, and an increase in the ECL with the increase in the reaction time was observed (Figure 3).

For array standardization, the amount of 8-oxodG in DNA oxidized by Fenton’s reagent was measured using UHPLC-MS/MS. The ratio of 8-oxodG to the total amount of dG increased with the oxidation time in the UHPLC-MS/MS experiments, which was used to obtain relative amounts of 8-oxodG. A calibration curve of %ECL increase vs. relative 8-oxodG concentration from UHPLC-MS/MS was used to quantitate 8-oxodG in the array (Figure 3d,e). The array was also used to measure metabolite-mediated DNA oxidation. This ECL array enabled 64 experiments in one run and only 1 µg of the DNA is required to perform an experiment. The detection limit was 1500 8-oxodG per 10^6^ nucleobases [40].

## 7. Summary and Conclusions

It is clear that multiple in vitro toxicity screening tools are needed to approach reliable predictions of in vivo toxicity risks. Our two-tiered approach of ECL microfluidic array and LC-MS/MS biocolloid reactors can provide detailed information on possible pathways of genotoxicity that are not forthcoming from bioassays, but are important components of a toxicity assessment. Microfluidic ECL arrays using RuPVP/DNA/enzyme films detect relative rates of DNA adduct damage from reactive metabolites. [Os(bpy)_2_(phen-benz-COOH)]^2+^ detects DNA oxidation. LC-MS/MS elucidates structures and measures the formation rates of individual nucleobase adducts and can also detect the amounts of 8-oxodG formed. Molecular pathway information obtained by these approaches can be integrated with other bioassays to provide better future predictions of genotoxicity of chemical and drug candidates.
